# Comparison of Hand Files, Mtwo, Reciproc, and Gentlefile Rotary Systems Regarding Canal Transportation, Centering Ability, and Obturation Quality of Primary Molars

**DOI:** 10.30476/dentjods.2024.100813.2250

**Published:** 2025-03-01

**Authors:** Maryam Kakoienejad, Mobina Najafifard, Sara Tavassoli-Hojjati, Ladan Hafezi, Saba Aghaei

**Affiliations:** 1 Dentist, Private Practice, Tehran, Iran; 2 Dept. of Pediatrics, Faculty of Dentistry, Tehran Medical Sciences, Islamic Azad University, Tehran, Iran; 3 Dept. of Radiology, Faculty of Dentistry, Tehran Medical Sciences, Islamic Azad University, Tehran, Iran

**Keywords:** Pulpectomy, Root Canal Obturation, Primary Teeth

## Abstract

**Statement of the Problem:**

Employing different instruments may have different impact on the outcome of root canal treatments. Deviation from the original canal path and defective root canal obturation may lead to pulpectomy treatment failure.

**Purpose:**

This study compared the primary molar canal transportation, centering ability, and obturation quality of hand files, Mtwo, Reciproc, and Gentlefile rotating machines in root canal treatments.

**Materials and Method:**

In this *in vitro* experimental study, eighty primary molar roots were randomly assigned to four groups. Cone-beam computed tomography (CBCT) scans were provided for the samples, and hand files (group 1), Mtwo (group 2), Reciproc (group 3), and Gentlefile (group 4) were used to instrument the root canals. Once more, CBCT scans were acquired, and at 1, 2, and 3mm from the apex as well as 1 mm from the orifice, the canal transportation and centering ability were evaluated in buccolingual and mesiodistal directions. Zeolite (ZOE) cement was used to obturate every root canal. To evaluate the obturation density, number of voids, and underfilling in each group, new CBCT scans were obtained. For every tooth, the maximum, minimum, and average Hounsfield units (HU) were noted. One-way ANOVA, the Kruskal-Wallis test, and Tukey's HSD test were used to analyze the data.

**Results:**

Mtwo exhibited considerably superior centering ability than Gentlefile at 2mm from the apex in the mesiodistal direction (*p* Value< 0.05). Gentlefile had significantly higher buccolingual canal transportation than Reciproc at 3 mm from the apex (P0.05). Minimum HU, underfilling, and void numbers did not differ amongst the four groups (*p*= 0.791, *p*= 0.1, and *p*= 0.548). Reciproc had substantially higher maximum and average HU, followed by Mtwo, Gentlefile, and hand files (*p*< 0.05).

**Conclusion:**

When compared to other systems, Gentlefile showed higher transportation and less centering ability. Reciproc had the highest obturation density, followed by Mtwo, Gentlefile, and hand files.

## Introduction

Pulpectomy is a successful method for preservation of primary teeth with irreversible pulpitis [ 1 ]. Root canal treatment is performed to completely obturate the root canal system and create a hermetic seal to prevent leakage of bacteria and toxic products into the periapical tissue [ 2 ]. Conical shaping of the root canal is imperative to enhance root canal irrigation and disinfection. It is also important to deliver root canal filling paste into the canal, and prevent overfilling, underfilling, and void formation [ 3 - 4 ]. Preservation of original canal shape and centering is an important criterion for optimal preparation of the root canal system. Deviation from the original canal path, especially at the apical region, can prevent optimal root canal obturation and sealing, leading to treatment failure [ 5 ]. Canal transportation is a common procedural error that may occur during root canal cleaning and shaping. In canal transportation, the root canal path is often deviated opposite to the direction of curvature, increasing the risk of root canal treatment failure, primary tooth loss, and subsequent permanent tooth space loss in dental arch [ 6 - 7 ]. Canal transportation in primary teeth is as important as that in permanent teeth [ 8 ]. Conventionally, stainless steel hand files are used for cleaning and shaping of primary teeth; however, due to their stiffness and low flexibility, they often cause procedural errors such as canal transportation [ 9 ]. Barr *et al.* [ 10 ] were the first to introduce rotary instruments for endodontic treatment in 2000. The advantages of rotary files over hand files include enhancement of root canal instrumentation, creation of smooth root canal walls in a shorter period of time, similarity with the root canal morphology, which leads to the preservation of root canal anatomy and curvature and decreases the incidence of iatrogenic errors especially in narrow and curved canals [ 1 , 11 ]. Reciproc and Gentlefile are two relatively new rotary systems. The Reciproc is a single nickel-titanium (NiTi)-file with reciprocating movement for root canal preparation [ 12 - 15 ]. These files have higher flexibility and resistance to cyclic fatigue than the conventional NiTi alloy [ 16 - 17 ]. The Gentlefile system includes stainless steel files, which are highly flexible due to their unique design, and can prepare the root canals with minimal pressure, maximal cleaning efficacy, and any cross-sectional design with unnecessary removal of tooth structure [ 18 - 20 ]. One drawback of NiTi rotary files is their inability to clean and shape the canals completely, such that approximately 35% of the canal surfaces remain unchanged after instrumentation with rotary files [ 21 ]. This is particularly important in primary teeth since they have different canal cross-sectional designs, accessory canals at the furcation area, fins and isthmi, and have a different internal geometry than permanent teeth [ 18 ]. The traditional experimental methods used for assessment of root filling and obturation quality include radiography, radioisotopes, coloring and staining, fluid filtration, bacterial leakage models, microscopic analysis, and clearing technique. However, none of these techniques could assess the obturation quality three-dimensionally [ 1 ]. The advent of cone beam computed tomography (CBCT) enabled reproducible three-dimensional assessment of volumes in dentistry without sectioning and material waste in addition to a significant lower effective dosage [ 22 - 23 ]. Previous studies evaluated canal transportation and centering ability in permanent teeth and reported conflicting results [ 7 , 18 , 24 - 25 ]. However, only one study [ 14 ] assessed the canal transportation in primary teeth by using WaveOne (reciprocating) and OneShape (rotary) systems. To the best of the authors’ knowledge, no previous study has compared the obturation quality of primary root canals instrumented with Mtwo, Reciproc, and Gentlefile systems. Thus, this study aimed to compare the obturation quality, canal transportation, and centering ability in primary teeth instrumented with hand files, Mtwo (VDW, Munich, Germany), Reciproc, and Gentlefile rotary systems *in vitro*.

## Materials and Method

This *in vitro*, experimental study was conducted on primary molars extracted due to periapical lesion or preventive orthodontic procedures. It was approved by Research Ethics Committee of Islamic Azad University, Dental Branch Tehran-Iran (IR.IAU.DENTAL.REC. 1397.043). The external surfaces of the roots were cleaned mechanically to remove calculus and soft tissue. The roots were disinfected in 0.5% sodium hypochlorite for 24 hours and stored in 0.1% distilled water and thymol solution until the experiment [ 26 ]. 

**Figure 1:**
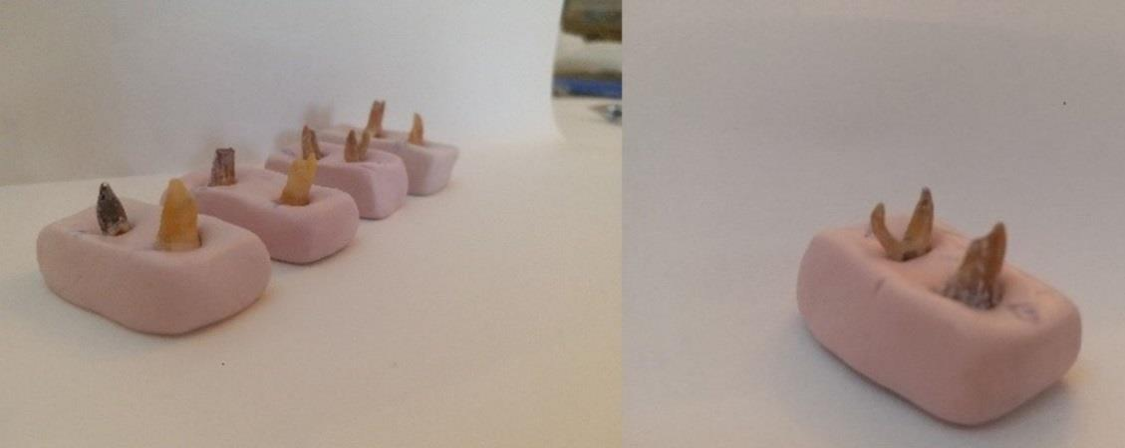
Mounting of the roots in putty to undergo cone beam computed tomography (CBCT)

The inclusion criteria were root canals of extracted primary molar teeth with 8-12 mm length, 5-10-degree curvature according to the Schneider’s method, internal dimensions of the canal equal to #25 K-file, and patency ensured with #10 K-file [ 27 ]. Roots with caries, cracks, and internal or external pathological resorption (as assessed on CBCT scans) were excluded. The sample size was calculated to be 20 in each group (a total of 80) for assessment of canal centering and transportation according to a study by Topcuglu *et al.* [ 8 ], assuming alpha= 0.05, beta=0.2, power=0.80, and effect size=0.33. The teeth were randomly assigned to the groups using a table of random numbers by Microsoft Excel 2021. The sample size was calculated to be 10 in each of the four groups for assessment of obturation quality according to a study by Deshpande *et al.* [ 4 ] assuming alpha=0.05, beta=0.2, mean standard deviation of the number of voids to be 1.2, and effect size of 0.5. The samples for obturation quality assessment were selected among the instrumented teeth for assessment of canal centering and transportation. After access cavity preparation, for standardization, the teeth were decoronated with a 009 diamond fissure bur (Jota, Switzerland) and high-speed hand-piece under water coolant such that the occlusogingival height of the pulp chamber was approximately 3mm from its floor [ 28 ]. The working length was determined 1 mm shorter than the length of a #10 K-file (Mani, Japan) when its tip was visible at the apex [ 29 ]. Putty impressions (Speedex, Switzerland) were then individually made from the teeth. After mixing of the impression material, the teeth were mounted in putty horizontally in a way that half the teeth was embedded in putty and their buccal surface was exposed and faced upwards. The longitudinal tooth axis was parallel to the longitudinal axis of the impressions (Figure 1). After assigning to the groups, the teeth underwent initial CBCT (NewTom, Giano, Italy) with a scanning time of 36 seconds, exposure time of 3-7 seconds, 60 kVp voltage, 3mA amperage, and 8×8 cm field of view. The slice thickness was 1mm (0.3mm voxel size). NNT Viewer version 2.21 was used to obtain images of sections perpendicular to the longitudinal axis of the canal (axial section) at four areas of 1, 2, and 3mm from the apex and 1mm from the orifice. The root canals were then instrumented by four methods of hand files (Mani, Japan), Mtwo (VDW, Munich, Germany), Reciproc (VDW, Munich, Germany), and Gentlefile (MedicNRG, Kibbutz Afikim, Israel). 

### Hand files

This group included 20 roots which were conventionally instrumented with hand K-files (Mani, Japan) and the standard technique with the following sequence: #15/0.02, #20/0.02, #25/0.02, and #30/0.02 [ 8 ].

### Mtwo rotary system

This group included 20 root canals, which were instrumented with Mtwo files (VDW, Munich, Germany) with gentle up-and-down movements and 280 rpm speed as instructed by the manufacturer with the following sequence: #10/0.04, #15/0.05, #20/0.06, #25/0.06, and #30/0.05 [ 8 ].

### Reciproc rotary system

The root canals (n=20) in this group were instrumented with Reciproc system (VDW, Munich, Germany), using R25 file with 25 mm cross-sectional area of the tip, and 8% taper using the Reciproc system endomotor (VDW Silver/Gold). The rubber stopper of the file was first adjusted to two-thirds of the working length, and the file was introduced into the canal with gentle pecking movement with a range not exceeding 3-4 mm. After reaching two-thirds of the working length and ensuring canal patency by using a #25 K-file. The rubber stopper of R25 file was adjusted to the working length, and its flutes were cleaned with a sterile gauze. It was then introduced into the canal again with gentle pecking movement to reach the working length. A final rinse (discussed as follows) was then performed as instructed [ 28 ].

### Gentlefile rotary system

The root canals (n=20) were instrumented with Gentle file system (MedicNRG, Kibbutz Afikim, Israel) in this group. GF1 file (22/0.04) was used with the respective hand-piece of this system operating at 6500 rpm. After using each file, the flutes were cleaned with a gauze dipped in alcohol. First, a #10 K-file was used to the working length to ensure apical patency. Next, GF1 was used with a pecking motion and gentle pressure for 5 seconds to reach the apical third of the canal. After reaching the working length, a final rinse was performed [ 28 ].

For all specimens, before and after using each file, the root canal was rinsed with 3 cc of sterile saline delivered via a 27-gauge needle which was passively inserted in the first 2 mm of the working length [ 30 ]. Each file was used for instrumentation of four root canals [ 28 ]. All instrumentations were performed by one operator. After root canal instrumentation, the roots were placed back in their putty impressions, and then they underwent CBCT again with the same exposure settings as those reported for the first CBCT. The provided scanning sections were reconstructed as explained for the initial CBCT and saved. 

Three teeth were used for assessment of the accuracy of scanner, impression technique, and standardization of position of imaging; they underwent primary and secondary CBCT examination with no root canal preparation. 

### Assessment of canal centering and transportation

Sections were reconstructed at 1, 2, and 3mm from the apex and 1mm from the orifice from primary (before instrumentation) and final (after instrumentation) CBCT scans by the related software. The distance between the external canal wall and the external root wall was measured at the mesial, distal, buccal, and lingual surfaces on before- and after-instrumentation images using the measurement tool of NNT Viewer software. The following formula was then used to calculate canal transportation and centering ability:

Canal transportation=(a1-a2)-(b1-b2)

**Figure 2:**
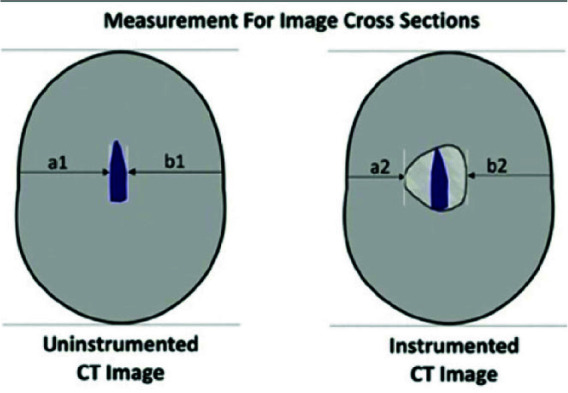
Schematic view of root cross-sections indicating a1, a2, b1, and b2 to calculate canal transportation and centering ability

**Figure 3:**
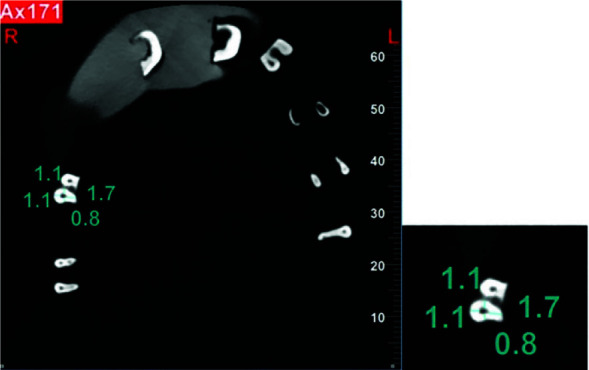
Measurement of mesial, distal, buccal, and lingual canal transportation using NNT software

Where a1 is the shortest distance between the distal (furcal) surface of the root and un-instrumented canal periphery, a2 is the shortest distance between the distal (furcal) surface of the root and instrumented canal periphery, b1 is the shortest distance between the mesial surface of the root and un-instrumented canal periphery, and b2 is the shortest distance between the mesial surface of the root and instrumented canal periphery (Figures 2-3).

According to the formula, a 0 result would indicate absence of canal transportation, positive values would indicate canal transportation towards the distal, and negative values would indicate canal transportation towards the mesial.

Centering ability was calculated using the following formula:

Centering ability = (a1-a2) / (b1-b2)

Accordingly, a result equal to 1 would indicate ideal centering; any other value would indicate sub-ideal centering ability. 

The same measurements were made in buccolingual direction. Information for each tooth was recorded in a datasheet. 

### Obturation quality

After root canal preparation, 10 root canals of each group were randomly selected by table of random numbers and dried with paper points and filled with zinc oxide eugenol (ZOE) cement (Kemdent, UK) using a #25 Lentulo spiral (Medin, Czech Republic) connected to a low-speed hand-piece in counterclockwise direction. The ZOE cement was prepared by mixing two scoops of powder with 2 drops of liquid as instructed by the manufacturer. The Lentulo spiral was dipped in ZOE and introduced into the canal to a certain length with rotational movements. Additional paste was gradually added until the canal was filled. Next, a moist cotton pellet was used to apply gentle pressure to pack the paste into the canal [ 2 ].

Subsequently, the teeth in all groups underwent CBCT to assess the obturation density and number of voids in each group. The teeth were placed back in their putty impressions (Speedex, Switzerland) and the obturation density was evaluated according to the CT number (CBCT Villa Italia) using OnDemand software. 

**Figure 4:**
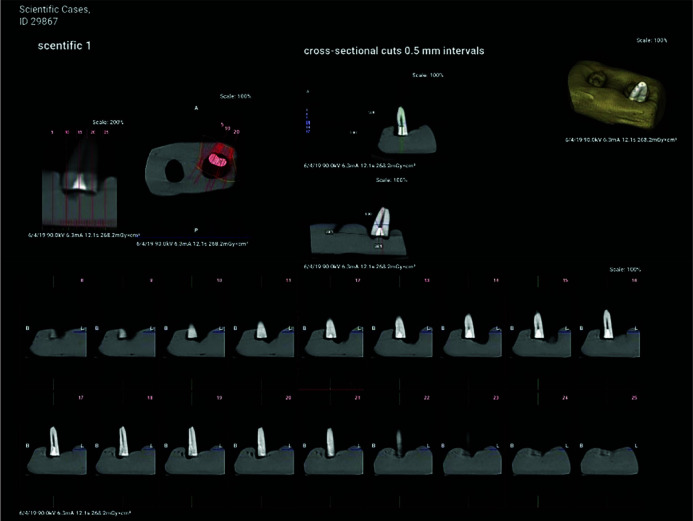
Standard reference CBCT to determine the Hounsfield units (HU) of zinc oxide eugenol (ZOE)

The reference standard for the CT number of ZOE was determined by using a 1×2mm box filled with ZOE mixed with the aforementioned powder/liquid ratio, which was placed on the coronal third of the root canal of a single-rooted primary tooth (such as a maxillary lateral incisor) with maximum condensation of material (to achieve the highest density) (Figure 4). 

**Figure 5:**
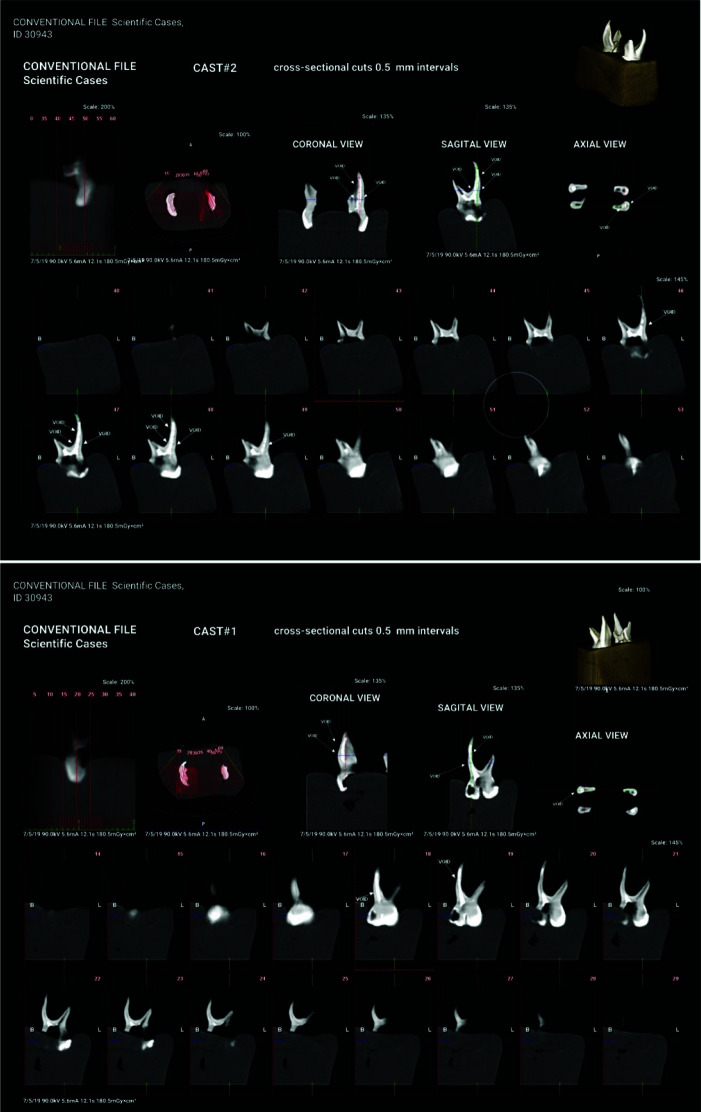
Cone beam computed tomography (CBCT) for assessment of root canal obturation quality

For each tooth, cross-sectional and axial sections were reconstructed with 0.5mm slice thickness and 0.5mm slice interval, and compared with the reference standard (Figure 5). The CT number and number of voids were calculated for each root from the cervical to the apical part of the root. The Hounsfield unit (HU) was used to calculate the CT number, which is a direct scale for measurement of density. It is based on the density of air (-1000 HU) and distilled water (0 HU) [ 31 ]. To assess the HU of specimens, 20 points were randomly selected from the coronal to apical part of the canal, and the HU of the selected points was recorded. For each root, the maximum HU, the minimum HU, and the average HU were recorded. The maximum, minimum, and average HU of the groups were compared with each other. Cross-sectional images with 0.5mm slice interval perpendicular to the root canal path were also reconstructed from axial sections. Accordingly, on each cross- sectional image of each root, the highest volume of root filling material and the largest surface area of the root were evaluated. In addition, root fillings shorter by over 1 mm from the apex were recorded as underfilled [ 32 ].

### Statistical analysis

Data of the four groups were analyzed by One-way AN-OVA test; the Tukey HSD test was applied for pairwise comparisons of the groups. Statistical analysis was conducted by SPSS version 25 and *p*< 0.05 was considered statistically significant.

## Results

### Canal centering and transportation

As shown in Table 1, mesial transportation was seen at 1mm from the apex in mesiodistal direction in all four groups, with no significant difference among them (*p*= 0.832). Centering ability was not significantly different among the groups either (*p*= 0.485).

**Table 1:** Comparison of canal transportation and centering ability at 1, 2, and 3 mm from the apex and 1 mm from the orifice in mesiodistal (MD) and buccolingual (BL) directions

Variable	1 mm from apex mean± SD				2 mm from apex mean± SD				3 mm from apex mean± SD				1 mm from orifice mean± SD			
System	Transportation		Centering Ability		Transportation		Centering Ability		Transportation		Centering Ability		Transportation		Centering Ability	
	MD	BL	MD	BL	MD	BL	MD	BL	MD	BL	MD	BL	MD	BL	MD	BL
Hand files	0.050±0.164-	0.075±0.137	0.285±0.713	0.592±1.027	0.060±0.135-	-0.015±0.150	0.250±0.716	0.100±0.447	-0.030±0.189	0.015±0.139	0.283±0.789	0.0125±0.319	0.005±0.143	-0.025±0.141	0.275±0.499	0.342±0.795
Reciproc	-0.055±0.13	0.040±0.150	0.200±0.696	0.150±0.671	-0.005±0.110	-0.015±0.278	0.547±0.707	0.350±0.933	0.050±0.173	0.080±0.330	0.325±0.730	0.312±0.567	0.010±0.215	-0.005±0.338	0.433±1.043	0.408±0.992
Mtwo	-0.015±0.21	0.020±0.191	0.617±1.630	0.250±0.618	0.020±0.219	0.040±0.226	0.627±1.095	0.575±1.389	-0.055±0.323	-0.040±0.119	0.596±0.762	0.253±0.504	-0.030±0.288	-0.045±0.204	0.900±1.901	0.162±0.400
Gentle file	-0.030±0.04	-0.040±0.12	0.200±0.410	0.262±0.367	-0.010±0.085	-0.065±0.150	0.000±0.000	0.200±0.410	0.015±0.131	-0.125±0.171	0.400±0.680	0.067±0.137	-0.035±0.184	-0.135±0.198	0.150±0.366	0.075±0.183
Overall *P* value	0.832	0.124	0.485	0.232	0.376	0.470	0.036	0.364	0.430	0.019	0.556	0.229	0.871	0.306	0.175	0.373

SD: Standard deviation

At 2mm from the apex in mesiodistal direction, mesial transportation was seen in Reciproc and Gentlefile while distal transportation was seen in Mtwo group; the difference among the groups was not significant (*p*= 0.376). Centering ability was zero in Gentlefile group (the lowest) and the highest in Mtwo group and this difference was significant (*p*< 0.05). 

At 3mm from the apex in mesiodistal direction, mesial transportation was noted in hand file and Mtwo groups, while distal transportation was seen in Reciproc and Gentlefile groups; the difference among the groups was not significant (*p*= 0.430). Centering ability was not significantly different among the groups either (*p*= 0.556). 

At 1mm from the orifice in mesiodistal direction, distal transportation was seen in hand file and Reciproc groups while mesial transportation was seen in Mtwo and Gentlefile groups; the difference among the groups was not significant (*p*= 0.871). Centering ability was not significantly different among the groups either (*p*= 0.175). 

Also, at 1mm from the apex in buccolingual direction, buccal transportation was seen in hand file, Reciproc, and Mtwo groups and lingual transportation was seen in Gentlefile group; the difference among the groups was not significant (*p*= 0.124). Centering ability was not significantly different among the groups either (*p*= 0.232).

At 2mm from the apex in buccolingual direction, all groups except Mtwo showed lingual transportation while Mtwo showed buccal transportation; the difference among the groups was not significant (*p*= 0.470). Centering ability was not significantly different among the groups either (*p*= 0.364). 

At 3mm from the apex in buccolingual direction, buccal transportation was noted in hand file and Reciproc groups while lingual transportation was noted in Mtwo and Gentlefile groups; the difference between Reciproc and Gentlefile was significant (*p*< 0.05). However, the centering ability of the groups was not significantly different (*p*= 0.229). 

At 1mm from the orifice in buccolingual direction, all groups showed lingual transportation; the difference among the groups was not significant (*p*= 0.306). Centering ability of the groups was not significantly different either (*p*= 0.373). 

### Obturation quality

As shown in Table 2, voids were present in all four groups with no significant difference among them (*p*= 0.548). The frequency of underfilling was not significantly different among the groups either (*p*= 0.1). Regarding density, the difference among the four groups was significant in maximum HU (*p*< 0.05), and average HU (*p*= 0.0001). The highest average HU was recorded in Reciproc group followed by Mtwo and Gentlefile, and finally the hand file group (*p*< 0.05). The difference among the four groups was not significant in minimum HU (*p*= 0.791). 

**Table 2:** Comparison of the number of voids, frequency of underfilling, and maximum, average, and minimum Hounsfield units (HU) in the study groups (*p*< 0.05 was considered statistically significant)

Variable	Number of voids Mean ± SD	Underfilling Mean ± SD	Max. HU Mean ± SD	Ave. HU Mean ± SD	Min. HU Mean ± SD
System					
Hand files N=10	1.90±2.846	1.350±1.76845	4220.00±1413.270	3370.00±759.093	2030.00±44.847
Mtwo N=10	1.30±1.337	1.0630±1.73643	6160.00±754.542	4120.00±277.088	2080.00±458.984
Reciproc N=10	1.70±1.767	0.00±0.000	8120.00±1047.537	4775.00±552.394	2190.00±467.737
Gentle N=10	0.70±0.823	0.3050±0.96449	5120.00±1074.761	3660.00±533.229	2200.00±418.994
Overall *P* value	0.548	0.1	0.0001	0.0001	0.791

SD: Standard deviation

HU: Hounsfield units

## Discussion

Several rotary systems have been proposed for root canal instrumentation of primary teeth; however, information regarding their efficacy, in comparison with hand files, is inconclusive and conflicting [ 32 ]. This experimental study aimed to compare obturation quality of hand files, Mtwo, Reciproc, and Gentlefile systems regarding the number of voids, obturation density, and underfilling, and canal transportation and centering ability in primary molars. The root canal anatomy and quality of instrumentation and obturation were assessed by CBCT. CBCT enables high quality and precise assessment of root canals before and after preparation by faster image acquisition and reconstruction, without requiring an examiner intervention [ 33 ]. It provides 3D images of different sections non-invasively and is as effective for primary teeth as for permanent teeth [ 1 ]. The present results showed that in all four groups, the root canals had mesial/distal and buccal/lingual transportation at 1, 2 and 3mm from the apex and 1mm from the orifice; no significant difference was found in canal transportation and centering ability among the four groups (*p*> 0.05) except for two comparisons. At 2mm from the apex in mesiodistal direction, the centering ability of Mtwo (0.627±1.095) was significantly higher than Gentlefile (0.000±0.000), and Mtwo preserved the canal centering significantly better than Gentlefile. At this level in mesiodistal direction, Mtwo had the highest and Gentlefile had the lowest centering ability. To the best of the authors’ knowledge, no previous study is available comparing the centering ability of Mtwo and Gentlefile, and information in this regard is scarce. Several studies compared Mtwo and Reciproc rotary files in highly curved permanent teeth and found no significant difference between them in canal transportation and centering ability [ 34 - 36 ]. The same results were obtained when comparing distal roots of primary mandibular first molars instrumented by Mtwo and Kedo-S. Haridoss *et al.* [ 37 ] reported that Mtwo had a good centering ability and cleaning efficiency for debris removal from curved canals. These NiTi files operate at 250-300 rpm and have an italic S-shaped cross-section with two cutting blades. They have a non-cutting tip, which enhances finding the correct path of the canal [ 38 ]. In addition, the presence of space between the blades in this file type, which gradually expand from the tip towards the shaft, prevents locking of the file in root canal walls during rotation [ 39 ]. A noteworthy issue is that the mean canal transportation in the present study, which was conducted on primary teeth, was higher than the mean value reported for permanent teeth using the same file systems [ 34 - 35 , 40 ]. One reason for this finding may be the different structure of dentin in primary and permanent teeth since primary dentin is softer than permanent dentin [ 41 ]. 

A significant difference in canal transportation was only noted at 3 mm from the apex in buccolingual direction between the Reciproc and Gentlefile groups. Canal transportation was greater in Gentlefile (-0.125±0.171) than Reciproc (0.0800±0.330). Several factors may be responsible for this difference. Reciproc has higher flexibility since it has undergone thermomechanical treatment and has a non-cutting tip, which decreases the risk of ledge formation and canal transportation [ 42 ]. Moreover, Reciproc has reciprocating movement. Thus, it has lower risk of locking in dentin and subsequent canal transportation, compared with Gentlefile with rotating (continuous rotation) movements [ 4 , 13 , 40 ]. The presence of a guiding tip in Reciproc, compared with Gentlefile, would guide the file into its correct path and prevent transportation; in addition, the speed of rotation of Reciproc is 300 rpm [ 43 ], while the speed of rotation of Gentlefile is 6500 rpm [ 18 ], which is several times higher than the speed of rotation of Reciproc. 

According to Fatah *et al.* [ 33 ], comparing dentin loss and canal transportation of primary teeth after instrumentation by two rotary systems, dentin loss in the mid-root and transportation in the apical area were significant. They attributed this finding to softer dentin structure at the apex due to dynamic phase of root resorption of primary teeth and transportation of the center of rotation in clockwise direction due to continuous rotation of the rotary system [ 33 ]. Another study compared the efficacy of different instruments for primary roots; they reported that hand files caused greater transportation in the apical region, showed higher frequency of lateral perforations in primary first and second molars, and resulted in less shaping of the canal, compared with ProTaper, and self-adjusting file. This finding may be due to canal preparation with #50 hand file in their study [ 44 ].

Due to the novelty of Gentlefile system, no study has compared its transportation or centering ability with hand files, Mtwo, or Reciproc. However, Saleh *et al.* [ 45 ], in 2018 compared canal transportation and centering ability of ProTaper Next NiTi rotary system and Gentlefile stainless steel rotary system and showed that Gentlefile caused significantly greater transportation at 3mm from the apex; however, the difference was not significant at 9 mm level. Their results were in agreement with the present findings. 

The present study appears to be the first to assess the quality of obturation by axial, coronal, sagittal, and cross-sectional CBCT images. HU is the scale for measurement of CT number (obturation density), which has not been used for evaluation of the quality of obturation of primary teeth so far. Accordingly, on each cross-sectional image of each root, the highest volume of root filling material and the largest surface area of the root were evaluated. Thus, the risk of bias due to the condensation of filling material in oblique sections was completely eliminated, and the HU of each section could be precisely compared with the HU of other sections and groups. Of different rotary systems, those with the highest resemblance to hand files in terms of adaptation to the canal walls, apical diameter, number of files, and technique of use were selected for the present study for the purpose of standardization as much as possible [ 28 ].

The results showed significantly higher average and maximum HU in the Reciproc group followed by Mtwo, Gentlefile, and hand files. The difference in minimum HU was not significant among the groups. The studies that compared the quality, volume, and density of primary root fillings reported higher obturation quality and density in canals instrumented with rotary systems, irrespective of file type, compared with hand files [ 3 , 4 , 9 , 32 , 46 - 47 ]. Since higher quality of obturation in rotary files, compared with hand files, may be due to better root canal instrumentation by rotary systems, it appears that the root canal shape after preparation with NiTi files is more conical, resulting in better delivery of root filling material into the canal [ 2 , 4 ]. Since roots with 8-12mm length were included in the present study, the orifice of the canals in the preparation process was at 8-12mm level from the file tip. The approximate cross-sectional diameter of Reciproc file at this length ranges from 0.88-1.05mm while that of Mtwo file is 0.50 to 0.70mm. Thus, the canal orifice after preparation in the Reciproc group would be larger than that in the Mtwo group. It appears that the increase in diameter results in a denser filling and enables easier delivery of root filling material into the canal. Thus, the Reciproc group showed the highest maximum HU, indicating maximum density of obturation. The cross-sectional diameter of the canal at this length is 0.54 to 0.70 mm for Gentlefile. The cross-sectional diameter of Mtwo and Gentlefile is the same; however, since the cross-sectional design of files probably affects their canal instrumentation efficacy [ 48 ], the S-shaped cross-sectional design and deep cutting blades of Mtwo [ 5 ] probably enhance easier delivery and better packing of ZOE in the canal, leading to higher obturation density in Mtwo, compared with Gentlefile group. 

The present results revealed the presence of voids and underfillings in all four groups, with no significant difference among them. This finding was in line with the results of Govindaraju *et al.* [ 49 ]. However, Panchal *et al.* [ 50 ] reported lower frequency of underfilling in Kedo-S compared with H-file and K-file groups. This difference may be attributed to the type of rotary system used in their study, which was a single-file system exclusive to primary teeth. It has a 12-mm cutting blade, and therefore, can better prepare the apical region [ 32 ]. Boonchoo *et al.* [ 47 ] evaluated primary mandibular molars and reported significantly higher frequency of underfilling in the mesial canal of the teeth in the hand file group. The reason may be the different filing movements, low elasticity of the files, and larger apical barrier due to greater extrusion of debris through the apex in this method that result in less preparation of the apical region. Overfilling was significantly more frequent in the mesial canals of the teeth in Wave 1 Reciproc rotary system group because in this system, preparation should be continued until the file becomes loose in the canal, which increases the risk of over-instrumentation [ 47 ]. Since root canals with specific criteria were included for standardization, the results may not be generalizable to narrower and curved root canals in primary teeth, and further investigations are required. In addition, equalization of the tip and taper size of the four systems were not possible since they are not specifically manufactured for the root canals of deciduous teeth. Therefore, future studies are suggested to assess newly introduced rotary systems in pediatric dentistry.

## Conclusion

Within the limitations of this study, results showed that all systems caused deviation from the original canal path towards mesial/distal and buccal/lingual, and none of the systems had ideal centering ability. No significant difference existed in canal transportation and centering ability among hand files, Mtwo, and Reciproc (*p*> 0.05). It appears that the Gentlefile system generally caused greater transportation and had lower centering ability compared with other systems. In addition, voids and underfilling were seen in all systems. Reciproc showed the highest obturation density followed by Mtwo, Gentlefile, and hand files. Considering easier and faster root canal preparation among the mentioned filling systems, Reciproc and Mtwo would probably lead to better canal anatomy preservation and obturation quality in wider canals of deciduous teeth.

## References

[1] Asokan S, Sooriaprakas C, Raghu V, Bairavi R ( 2012). Volumetric analysis of root canal fillings in primary teeth using spiral computed tomography: an in vitro study. J Dent Child (Chic)..

[2] Kumar S, Raj S, Konde S, Rai K ( 2016). Comparison of obturation techniques using three delivery systems: an in vitro study. MJDS.

[3] Govindaraju L, Jeevanandan G, Subramanian E ( 2017). Clinical evaluation of quality of obturation and instrumentation time using two modified rotary file systems with manual instrumentation in primary teeth. J Clin Diagn Res.

[4] Deshpande AN, Joshi NH, Naik KS ( 2017). In vitro comparative evaluation of cleaning efficacy and volumetric filling in primary molars: cone beam computed tomography evaluation. Contemp Clin Dent.

[5] Kardon BP, Kuttler S, Hardigan P, Dorn SO ( 2003). An in vitro evaluation of the sealing ability of a new root-canal-obturation system. J Endod.

[6] Yoshimine Y, Ono M, Akamine A ( 2005). The shaping effects of three nickel-titanium rotary instruments in simulated S-shaped canals. J Endod.

[7] Jafarzadeh M, Saatchi M, Jafarnejadi P, Gooran M ( 2019). Digital radiographic evaluation of the quality of different root canal obturation techniques in deciduous mandibular molars after preparation with rotary technique. J Dent Shiraz Univ Med Sci.

[8] Topçuoğlu G, Topçuoğlu HS, Akpek F ( 2016). Evaluation of apically extruded debris during root canal preparation in primary molar teeth using three different rotary systems and hand files. Int J Paediatr Dent.

[9] Makarem A, Ravandeh N, Ebrahimi M ( 2014). Radiographic Assessment and Chair Time of Rotary Instruments in the Pulpectomy of Primary Second Molar Teeth: A Randomized Controlled Clinical Trial. J Dent Res Dent Clin Dent Prospects.

[10] Barr ES, Kleier DJ, Barr NV ( 2000). Use of nickel-titanium rotary files for root canal preparation in primary teeth. Pediatr Dent.

[11] Pinheiro SL, Neves LS, Imparato JCP, Duarte DA, Bueno CES, Cunha RS ( 2012). Analysis of the instrumentation time and cleaning between manual and rotary techniques in deciduous molars. RSBO (Online)..

[12] Gungor OE, Kustarci A ( 2016). Evaluation of Apically Extruded Debris using Two Niti Systems Associated with Two İrrigation Techniques in Primary Teeth. J Clin Pediatr Dent.

[13] Ramazani N, Mohammadi A, Amirabadi F, Ramazani M, Ehsani F ( 2016). In vitro investigation of the cleaning efficacy, shaping ability, preparation time and file deformation of continuous rotary, reciprocating rotary and manual instrumentations in primary molars. J Dent Res Dent Clin Dent Prospects.

[14] Prabhakar AR, Yavagal C, Dixit K, Naik SV ( 2016). Reciprocating vs Rotary Instrumentation in Pediatric Endodontics: Cone Beam Computed Tomographic Analysis of Deciduous Root Canals using Two Single-file Systems. Int J Clin Pediatr Dent.

[15] Nouroloyouni A, Shahi S, Salem Milani A, Noorolouny S, Farhang R, Yousefi Azar A ( 2023). In vitro apical extrusion of debris and instrumentation time following root canal instrumentation with Reciproc and Reciproc Blue instruments and a novel stainless steel rotary system (Gentlefile) versus manual instrumentation. J Dent Res Dent Clin Dent Prospects.

[16] Larsen CM, Watanabe I, Glickman GN, He J ( 2009). Cyclic fatigue analysis of a new generation of nickel titanium rotary instruments. J Endod.

[17] Ye J, Gao Y ( 2012). Metallurgical characterization of M-Wire nickel-titanium shape memory alloy used for endodontic rotary instruments during low-cycle fatigue. J Endod.

[18] Moreinos D, Dakar A, Stone NJ, Moshonov J ( 2016). Evaluation of Time to Fracture and Vertical Forces Applied by a Novel Gentlefile System for Root Canal Preparation in Simulated Root Canals. J Endod.

[19] Nouroloyouni A, Safavi Hir F, Farhang R, Noorolouny S, Salem Milani A, Alyali R ( 2023). Evaluating in vitro performance of a novel stainless steel rotary system (Gentlefile) based on debris extrusion and instrumentation Time. Biomed Res Int.

[20] Godiny M, Mohammadi B, Norooznezhad M, Chalabi M ( 2023). Endodontic rotary systems: comparison between Gentlefile and pro taper universal for removal of enterococcus faecalis. J Clin Exp Dent.

[21] Peters OA, Schönenberger K, Laib A ( 2001). Effects of four Ni-Ti preparation techniques on root canal geometry assessed by micro computed tomography. Int Endod J.

[22] Nouroloyouni A, Salem Milani A, Etminan A, Noorolouny S, Tavakkol E, Mikaieli Xiavi H, et al ( 2023). Cone-beam computed tomography assessment of quality of endodontic treatment and prevalence of procedural errors in mandibular molars. Int J Clin Pract.

[23] Nouroloyouni A, Basser R, Salehi Z, Farhang R, Zadfattah F, Aghajani M ( 2019). Evaluating the iatrogenic errors and the quality of root canal treatment of mandibular premolars in Ardabil population using the cone beam computed tomography in 2018. Avicenna J Dent Res.

[24] Kucukyilmaz E, Savas S, Saygili G, Uysal B ( 2015). Evaluation of apically extruded debris and irrigant produced by different Nickel-Titanium instrument systems in primary teeth. J Contemp Dent Pract.

[25] Nazari Moghaddam K, Mehran M, Farajian Zadeh H ( 2009). Root canal cleaning efficacy of rotary and hand files instrumentation in primary molars. Iran Endod J.

[26] Labbaf H, Shakeri L, Orduie R, Bastami F ( 2015). Apical extrusion of debris after canal preparation with hand-files used manually or installed on reciprocating air-driven handpiece in straight and curved canals. Iran Endod J.

[27] Schneider SW ( 1971). A comparison of canal preparations in straight and curved root canals. Oral Surg Oral Med Oral Pathol.

[28] Bahmani P, Aghaei S, Tavassoli-Hojjati S, Labbaf H ( 2023). A comparison of apically extruded debris and root canal preparation time of primary mandibular second molars using hand files, Mtwo, Reciproc, and Gentlefile rotary systems: An in vitro study. Neuro Quantology.

[29] Ebrahimzade E, Tavassoli Hojjati S, Aghaei S ( 2021). Comparison of apically extruded debris and instrumentation time using rotary, endodontic handpiece and hand files In primary molar teeth: in vitro. J Res Dent Sci.

[30] Dentistry AAOP ( 2023). Pulp therapy for primary and immature permanet teeth. The reference manual of pediatric dentistry. Chicago, III. American Academy of Pediatric Dentistry.

[31] Cassetta M, Stefanelli LV, Pacifici A, Pacifici L, Barbato E ( 2014). How accurate is CBCT in measuring bone density? A comparative CBCT-CT in vitro study. Clin Implant Dent Relat Res.

[32] Jeevanandan G, Govindaraju L ( 2018). Clinical comparison of Kedo-S paediatric rotary files vs manual instrumentation for root canal preparation in primary molars: a double blinded randomised clinical trial. Eur Arch Paediatr Dent.

[33] Fatah YAM, Khattab NMA, Gomaa YF, Elheeny AH ( 2022). Cone-beam computed tomography analysis of primary root canals transportation and dentin loss after instrumentation with two-pediatric rotary files. BMC Oral Health.

[34] Navós BV, Hoppe CB, Mestieri LB, Böttcher DE, Reis Só MV, Grecca FS ( 2016). Centering and transportation: in vitro evaluation of continuous and reciprocating systems in curved root canals. J Conserv Dent.

[35] Mamede-Neto I, Borges AH, Guedes OA, Oliveira D, Pedro FLM, Estrela C ( 2017). Root canal transportation and centering ability of nickel-titanium rotary instruments in mandibular premolars assessed using cone-beam computed tomography. Open Dent J.

[36] Mesgarani A, Hamidi MR, Haghanifar S, Naiemi S, Ali Bijani A ( 2018). Comparison of apical transportation and centering ability of Mtwo and Reciproc R25 in severely curved canals using cone-beam computed tomography. Dent Res J (Isfahan)..

[37] Haridoss S, Rakkesh KM, Swaminathan K ( 2022). Transportation and centering ability of Kedo-S pediatric and Mtwo instruments in primary teeth: a cone-beam computed tomography Study. Int J Clin Pediatr Dent.

[38] Hamze F, Honardar K, Nazarimoghadam K ( 2011). Comparison of two canal preparation techniques using Mtwo rotary instruments. Iran Endod J.

[39] VDW Gold reciproc: endo motor with integrated length determination. http://www.vdw-dental.com.

[40] Jainaen A, Mahakunakorn N, Arayatrakullikit U, Sutthiprapaporn P, Noisombat R ( 2018). Cone-beam computed tomography evaluation of curved root canals prepared using reciprocal rotary files and rotational rotary files. J Conserv Dent.

[41] George S, Anandaraj S, Issac JS, John SA, Harris A ( 2016). Rotary endodontics in primary teeth: a review. Saudi Dent J.

[42] Shen Y, Zhou HM, Zheng YF, Peng B, Haapasalo M ( 2013). Current challenges and concepts of the thermomechanical treatment of nickel-titanium instruments. J Endod.

[43] Kim HC, Kwak SW, Cheung GS, Ko DH, Chung SM, Lee W ( 2012). Cyclic fatigue and torsional resistance of two new nickel-titanium instruments used in reciprocation motion: reciproc versus wave one. J Endod.

[44] Hidalgo LRC, Silva LAB, Leoni GB, Mazzi-Chaves JF, Carvalho EES, Consolaro A, et al ( 2017). Mechanical preparation showed superior shaping ability than manual technique in primary aolars: A micro-computed tomography study. Braz Dent J.

[45] Saleh MA, Zaazou AM, Leheta NA ( 2018). Evaluation of canal transportation and centring ability of nickel-titanium versus stainless steel rotary systems: an in vitro study. Endod Prac.

[46] Poornima P, Disha P, Nagaveni NB, Roopa KB, Bharath KP, Neena IE ( 2016). Volumetric analysis of hand and rotary root canal instrumentation and filling in primary teeth using spiral computed tomography: an in vitro study. Int J Paediatr Dent.

[47] Boonchoo K, Leelataweewud P, Yanpiset K, Jirarattanasopha V ( 2020). Simplify pulpectomy in primary molars with a single-file reciprocating system: a randomized controlled clinical trial. Clin Oral Investig.

[48] Jalali S, Eftekhar B, Paymanpour P, Yazdizadeh M, Jafarzadeha M ( 2015). Effects of Reciproc, Mtwo and ProTaper instruments on formation of root fracture. Iran Endod J.

[49] Govindaraju L, Jeevanandan G, Subramanian EMG ( 2017). Comparison of quality of obturation and instrumentation time using hand files and two rotary file systems in primary molars: a single-blinded randomized controlled trial. Eur J Dent.

[50] Panchal V, Jeevanandan G, Subramanian E ( 2019). Comparison of instrumentation time and obturation quality between hand K-file, H-files, and rotary Kedo-S in root canal treatment of primary teeth: a randomized controlled trial. J Indian Soc Pedod Prev Dent.

